# Supplementary material for: Total Polyunsaturated Fatty Acid Level in Abdominal Adipose Tissue as an Independent Predictor of Recurrence-Free Survival in Women with Ovarian Cancer
Source: Int J Mol Sci. 2023 Jan 16;24(2):1768. doi: 10.3390/ijms24021768 (PMC9863501; doi:10.3390/ijms24021768)
Supplement: Supplementary file 1 [file ijms-24-01768-s001.zip › ijms-2042337-supplementary.pdf]

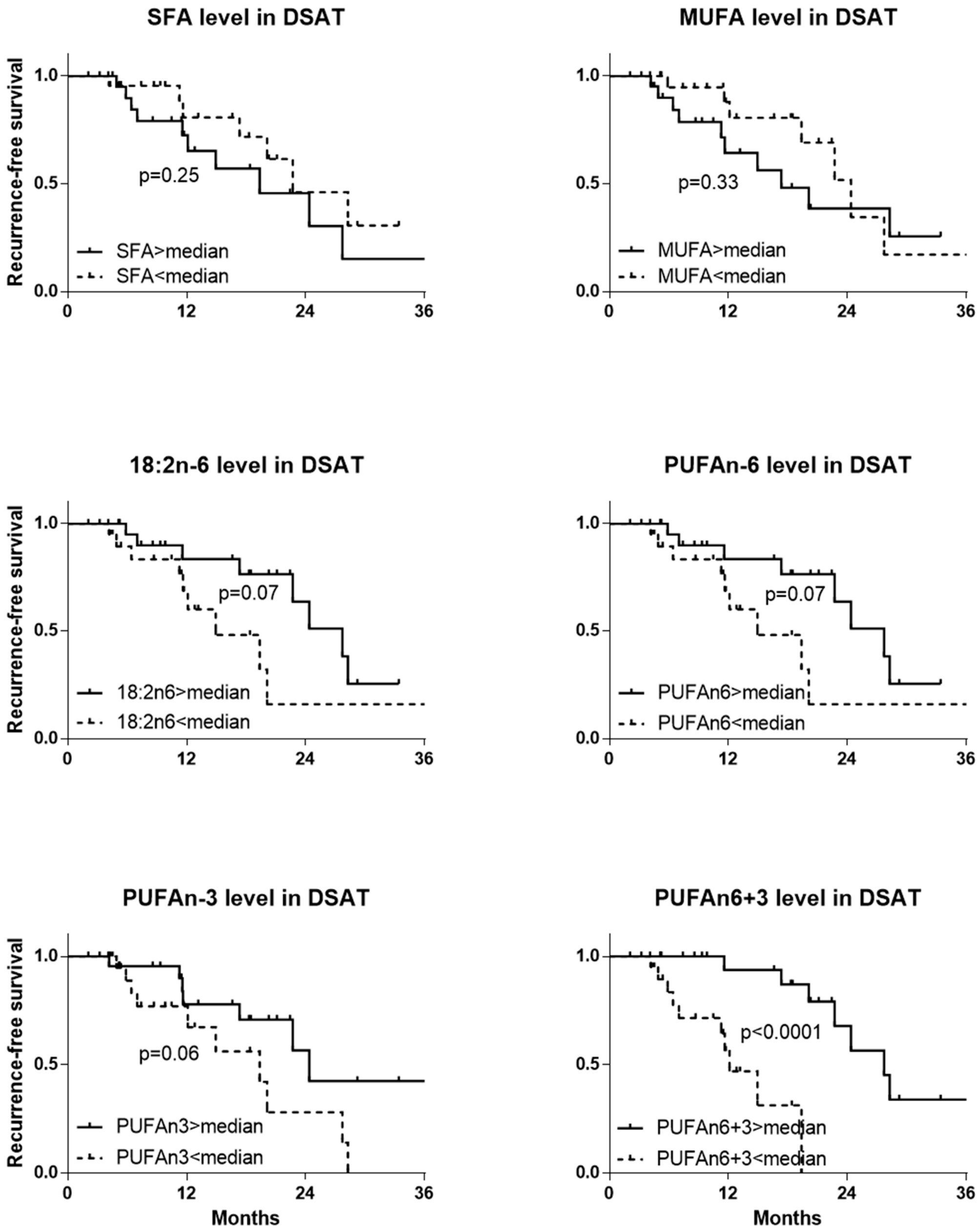
SFA level in DSAT
Recurrence-free survival
p=0.25
SFA>median
SFA<median
MUFA level in DSAT
p=0.33
MUFA>median
MUFA<median
18:2n-6 level in DSAT
p=0.07
18:2n6>median
18:2n6<median
PUFAn-6 level in DSAT
p=0.07
PUFAn6>median
PUFAn6<median
PUFAn-3 level in DSAT
p=0.06
PUFAn3>median
PUFAn3<median
PUFAn6+3 level in DSAT
p<0.0001
PUFAn6+3>median
PUFAn6+3<median
Months

(**A**)

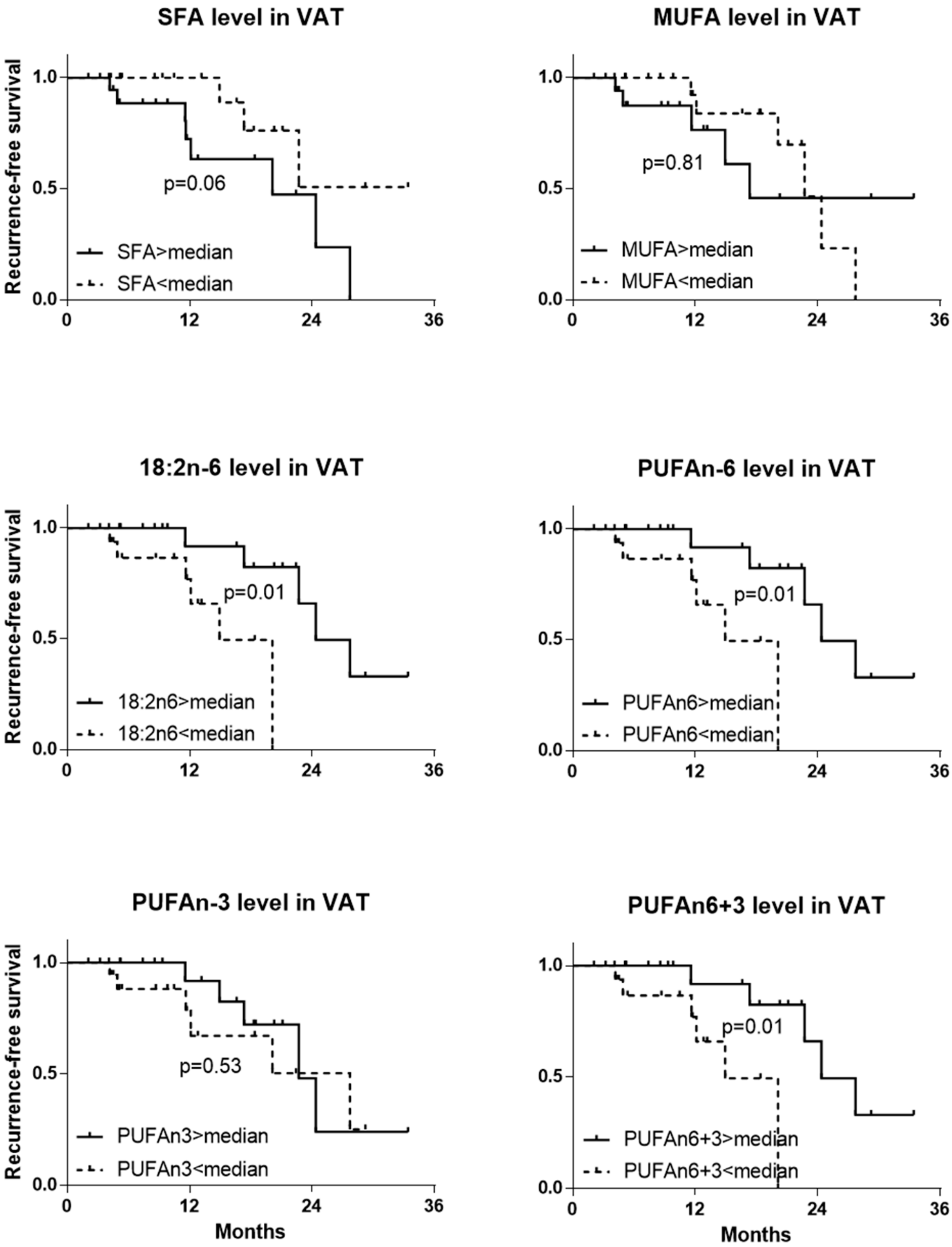
SFA level in VAT
Recurrence-free survival
p=0.06
SFA>median
SFA<median
MUFA level in VAT
p=0.81
MUFA>median
MUFA<median
18:2n-6 level in VAT
p=0.01
18:2n6>median
18:2n6<median
PUFAn-6 level in VAT
p=0.01
PUFAn6>median
PUFAn6<median
PUFAn-3 level in VAT
p=0.53
PUFAn3>median
PUFAn3<median
Months
PUFAn6+3 level in VAT
p=0.01
PUFAn6+3>median
PUFAn6+3<median
Months

(**B**)

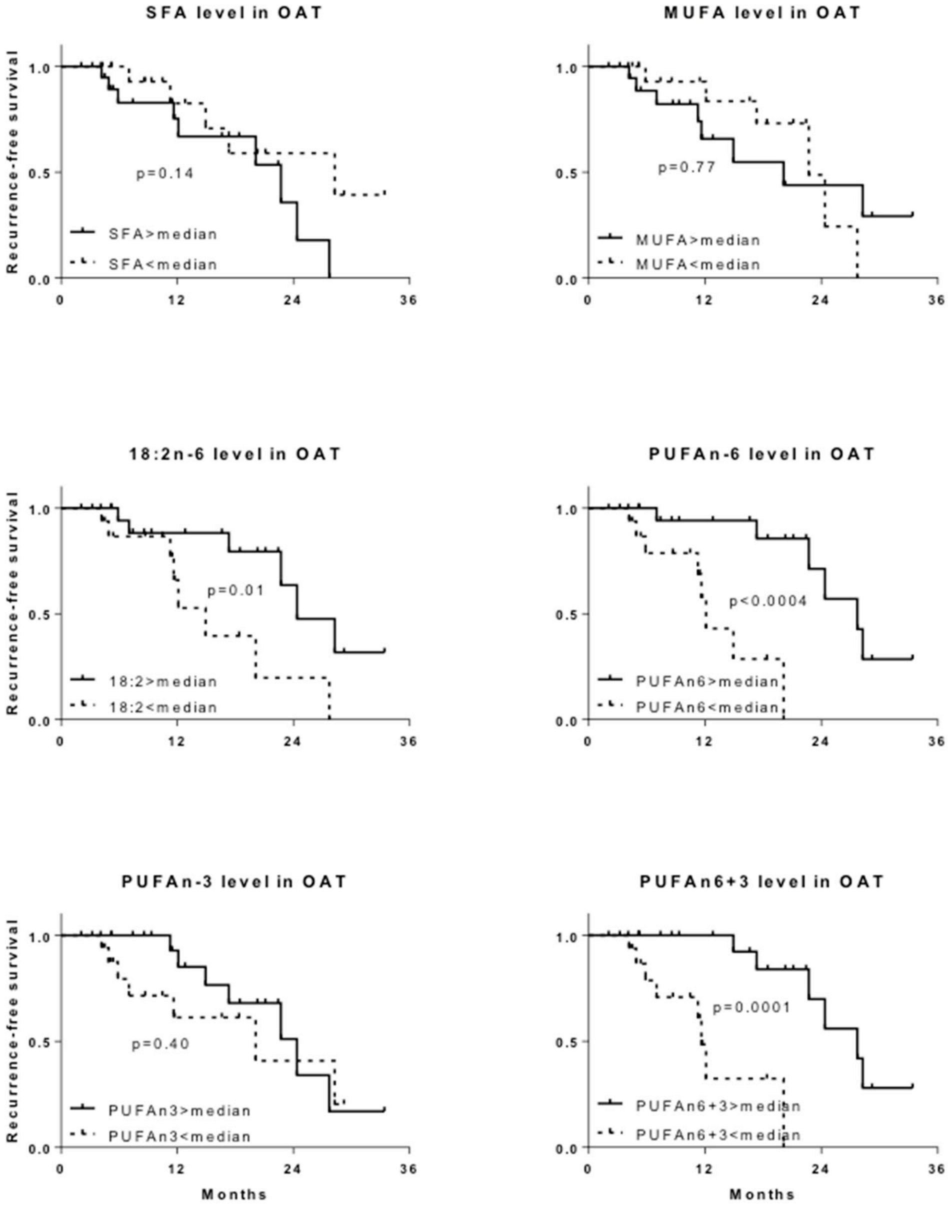

(**C**)

Supplementary Figure S1. (**A**): Recurrence-free-survival (RFS) in patients with epithelial ovarian cancer (EOC) by fatty acid content in deep subcutaneous adipose tissue (DSAT). Analysis of recurrence-free survival by the Kaplan-Meier method and LogRank test. Women were divided into 2 groups by fatty acid content in deep subcutaneous adipose tissue (< median, n=23 or > median, n=23), n=46. SFAs: saturated fatty acids, MUFAs: monounsaturated fatty acids, PUFAs: polyunsaturated fatty acids, DSAT: Deep subcutaneous adipose tissue, 18:2n-6: linoleic acid; (**B**): Recurrence-free survival in patients with EOC by fatty acid content in visceral adipose tissue (VAT). Analysis of RFS with Kaplan-Meier method and LogRank test. Patients were divided into 2 groups according to fatty acid content in epiploic adipose tissue (<median, n=19 or > median, n=19), n=38. SFA: saturated fatty acid, MUFA: monounsaturated fatty acid, PUFA: polyunsaturated fatty acid, EAT: Epiploic adipose tissue, 18:2n-6: linoleic acid. (**C**) Recurrence-free survival in patients with EOC by fatty acid levels in omental adipose tissue (OAT). Analysis of RFS with Kaplan-Meier method and LogRank test. Patients were divided into 2 groups by fatty acid content in omental adipose tissue (< median, n=19 or ≥ median, n=20), n=39. SFA: saturated fatty acid, MUFA: monounsaturated fatty acid, PUFA: polyunsaturated fatty acid, OAT: Omentum adipose tissue, 18:2n-6: linoleic acid.
